# Comprehensive quantitative sensory testing shows altered sensory function in women with chronic pelvic pain: results from the Translational Research in Pelvic Pain (TRiPP) Study

**DOI:** 10.1097/j.pain.0000000000002955

**Published:** 2023-06-07

**Authors:** Lydia Coxon, Jan Vollert, Danielle Perro, Claire E. Lunde, Joana Ferreira-Gomes, Ana Charrua, Pedro Abreu-Mendes, Michal Krassowski, Judy Birch, Jane Meijlink, Lone Hummelshoj, Anja Hoffmann, Qasim Aziz, Lars Arendt-Nielsen, Esther Pogatzki-Zahn, Emma Evans, Lysia Demetriou, Stephen B. McMahon, Stacey A. Missmer, Christian M. Becker, Krina T. Zondervan, Andrew W. Horne, Francisco Cruz, Christine B. Sieberg, Rolf-Detlef Treede, Jens Nagel, Katy Vincent

**Affiliations:** a Nuffield Department of Women's and Reproductive Health, University of Oxford, Oxford, United Kingdom; b University Hospital Muenster, Muenster, Germany; c Heidelberg University, Mannheim, Germany; d Pain Research, Department of Surgery and Cancer, Imperial College London, London, United Kingdom; e Division of Neurological Pain Research and Therapy, Department of Neurology, University Hospital of Schleswig-Holstein, Campus Kiel, Germany; f Biobehavioral Pain Innovations Lab, Department of Psychiatry & Behavioral Sciences, Boston Children's Hospital, Boston, MA, United States; g Pain and Affective Neuroscience Center, Department of Anesthesiology, Critical Care, and Pain Medicine, Boston Children's Hospital, Boston, MA, United States; h IBMC/I3S, Faculty of Medicine of Porto & Hospital São João, Porto, Portugal; i Pelvic Pain Support Network, Poole, United Kingdom; j International Painful Bladder Foundation, Naarden, the Netherlands; k Endometriosis.org, London, United Kingdom; l Bayer AG, Research & Development, Pharmaceuticals, Berlin, Germany; m Queen Mary University of London, London, United Kingdom; n Center for Neuroplasticity and Pain (CNAP), SMI, Department of Health Science and Technology, Aalborg University, Aalborg, Denmark; o Department of Medical Gastroenterology, Mech-Sense, Aalborg University Hospital, Aalborg, Denmark; p Formerly of Neurorestoration Group, Wolfson Centre for Age-Related Diseases, King's College London, London, United Kingdom; q Department of Obstetrics, Gynecology, and Reproductive Biology, College of Human Medicine, Michigan State University, Grand Rapids, MI, United States; r Department of Epidemiology, Harvard T.H. Chan School of Public Health, Boston, MA, United States; s Division of Adolescent and Young Adult Medicine, Department of Pediatrics, Boston Children's Hospital and Harvard Medical School, Boston, MA, United States; t University of Edinburgh, Edinburgh, United Kingdom; u Department of Psychiatry, Harvard Medical School, Boston, MA, United States; v Bayer AG, Research & Development, Pharmaceuticals, Wuppertal, Germany

**Keywords:** Quantitative sensory testing, Chronic pelvic pain, Interstitial cystitis, Bladder pain syndrome, Endometriosis

## Abstract

Chronic pelvic pain (CPP), despite its high prevalence, is still relatively poorly understood mechanistically. This study, as part of the Translational Research in Pelvic Pain (TRiPP) project, has used a full quantitative sensory testing (QST) paradigm to profile n = 85 women with and without CPP (endometriosis or bladder pain specifically). We used the foot as a control site and abdomen as the test site. Across 5 diagnostically determined subgroups, we found features which are common across different aetiologies, eg, gain of function in pressure pain threshold (PPT) when assessing responses from the lower abdomen or pelvis (referred pain site). However, disease-specific phenotypes were also identified, eg, greater mechanical allodynia in endometriosis, despite there being large heterogeneities within diagnostic groups. The most common QST sensory phenotype was mechanical hyperalgesia (>50% across all the groups). A “healthy’ sensory phenotype was seen in <7% of CPP participants. Specific QST measures correlated with sensory symptoms assessed by the painDETECT questionnaire (pressure-evoked pain [painDETECT] and PPT [QST] [*r* = 0.47, *P* < 0.001]; mechanical hyperalgesia (painDETECT) and mechanical pain sensitivity [MPS from QST] [*r* = 0.38, *P* = 0.009]). The data suggest that participants with CPP are sensitive to both deep tissue and cutaneous inputs, suggesting that central mechanisms may be important in this cohort. We also see phenotypes such as thermal hyperalgesia, which may be the result of peripheral mechanisms, such as irritable nociceptors. This highlights the importance of stratifying patients into clinically meaningful phenotypes, which may have implications for the development of better therapeutic strategies for CPP.

## Abstract 2

Quantitative sensory testing in chronic pelvic pain shows changes to sensation, including deep tissue and cutaneous inputs, suggesting central and peripheral mechanisms may be important.

## 1. Introduction

Chronic pelvic pain (CPP) affects between 5% and 26.6% of women worldwide.^1,19,77,78^ Despite the high prevalence of CPP, there is still little understanding of the underlying mechanisms giving rise to and maintaining pain in these women. The Translational Research in Pelvic Pain (TRiPP) study is a collaboration across sites in the United Kingdom, Europe, and United States which focusses specifically on endometriosis and interstitial cystitis or bladder pain syndrome (IC/BPS),^15^ with an overall aim of better understanding the mechanisms underlying CPP in women.^16^ Given the increasing evidence of similarities between CPP and other chronic pain conditions, we have focused on both local pelvic and systemic or central mechanisms.

Quantitative sensory testing (QST) is a psychophysical method of testing the function of the somatosensory nervous system.^13^ Individual somatosensory profiles including “sensory loss” and “sensory gain” have been shown to vary in different aetiologies and on an individual basis for the same diseases.^6,13,41^ To date, detailed QST profiling has not been performed in women with CPP, although many studies have used single sensory modalities.^3,28,30,31,56,75^ We used the QST paradigm developed by the German Research Network on Neuropathic Pain (DFNS),^40,54^ comprising a series of standardised tests to assess the detection and pain thresholds for different types of stimuli. These relate to specific neuroanatomical pathways with separate nerve fibre populations. In addition to characterising the sensory profiles associated with specific pathologies, it has also been shown that, across aetiologies, distinct clusters of sensory profiles can be found.^8,70^ Four subgroups can be defined using a published algorithm^70^ representing (1) “sensory loss” (mechanical and thermal sensory loss), (2) “thermal hyperalgesia” (preserved sensory function with heat or cold hyperalgesia), (3) “mechanical hyperalgesia” (loss of thermal sensation with mechanical hyperalgesia or allodynia), and (4) “healthy” (sensation similar to the pain-free population).^8^ By profiling of human surrogate models, these profiles can be tentatively taken as evidence supporting denervation and peripheral and central sensitisation.^69^

The aim of this study was to apply the DFNS QST profiling tool to assess sensory phenotypes in women with CPP. We hypothesise that many women with CPP have altered sensory profiles similar to other chronic pain conditions, whereas some features may be specific to their underlying diagnosis and aetiology. Given that the DFNS QST protocol is time consuming, we also aim to determine whether some of the QST measures correlate with scores from the painDETECT questionnaire,^23,24^ a brief self-report tool that could easily be integrated into clinical practice if shown to be of value in assessing underlying pain mechanisms.

## 2. Methods

### 2.1. Participant recruitment

Three sites participated in the study: University of Oxford, UK (OX), Boston's Children's Hospital, USA (BCH) & Instituto de Biologia Molecular e Celular, Portugal (IBMC). Participants from OX and BCH were selected based on criteria from an existing database of participants from parent studies. Participants from IBMC were recruited through urology clinics.^15^ All appropriate ethical approvals were secured before recruitment into TRiPP (ethics reference 19/YH/0030).

Recruitment was restricted to females aged 18 to 50 who were neither pregnant nor lactating. Participants were recruited into 1 of 5 groups: endometriosis-associated pain (EAP) who have previously received a surgical diagnosis of endometriosis and at least one type of pelvic pain >4/10; endometriosis-associated pain with comorbid bladder pain (EABP) who meet EAP criteria with additional pain experienced in the bladder and urinary symptoms (urinary frequency and/or urgency symptoms)^45^; bladder pain syndrome (BPS) who fit the clinical presentation of pain perceived to arise from the bladder >4/10 and urinary symptoms (urinary frequency and/or urgency), with no previous surgical diagnosis of endometriosis^45^; pelvic pain without bladder pain or urinary symptoms and no previous surgical diagnosis of endometriosis (PP), and pain-free controls (CON). In summary, participants were either newly contacted and recruited (IBMC) or contacted after participation in a “parent” study (EndOX: A study to identify possible biomarkers in women with endometriosis, Oxford REC ref:09/H0604/58; Boston Center for Endometriosis [BCE]: A Cross-Institutional Biorepository and Database, IRB-P00004267) and then recruited into the current study (OX and BCH), more detailed information on participant criteria and study protocol can be seen in Demetriou et al.^15^ Participants had undergone surgery where this was a component of the standard of care. This includes all participants with an endometriosis diagnosis (EAP and EABP) and also all other participants from OX where recruitment was only from clinics. Most participants recruited from the community at BCH and from Urology clinics in IBMC had not had surgery; however, any potential participant in the BPS or PP groups who had had endometriosis identified previously was excluded. More details of the surgical history and where relevant endometriosis staging are given in Table 2. There were no recruitment criteria based on the duration of chronic pain or number of days participants experienced pain per month. Table 2 shows the duration of pain in years for each of the study groups. Pain was not exclusive to the menstrual cycle, with participants reporting noncyclical pelvic pain, dyspareunia, dyschezia, and dysuria (as can be seen in another TRiPP manuscript illustrating the cohort).^16^

All participants gave informed consent. Data were collected between January 2020 and August 2021. All participants from IBMC had study visits and questionnaires completed in Portuguese. Validated Portuguese versions of study material was used where available, otherwise material was forward and backward translated. Unfortunately, all control participants were recruited at Boston; therefore, it is not possible to determine the effect of site in this cohort, although previous studies have shown little or no impact of the site of data collection on QST measures.^68^

### 2.2. Study visit

After coordinated training of all experimenters, the DFNS QST protocol was performed on the dorsum of the right foot (control site) and the lower abdomen or pelvis (test site).

All QST sessions were performed in a temperature-controlled room at approximately 20°C. Participants were also asked to complete a battery of questionnaires, which included painDETECT^23,24^ as detailed in our protocol.^15^ Before the session, participants were also asked to complete a “How are you today?” questionnaire assessing factors that may impact on psychophysical measures: current pain intensity (Numerical Rating Scale [NRS] 0 to 10), state anxiety,^58^ pain catastrophizing,^61^ medication and caffeine use that day, and day of menstrual cycle.

The QST script was translated into Portuguese for participants at IBMC. The QST script was forward and back translated before use in this study as no published script was available. A previous study has addressed issues of translating QST scripts into other European languages (not Portuguese, though) and found that it seems possible to produce highly reliable DFNS QST results across different research units, and, more importantly, also across countries and languages.^68^

Quantitative sensory testing was performed by trained researchers who had undergone training with DFNS in Mannheim (September 2019) or attended a virtual training refresher session before recommencing data collection after the peak of the COVID-19 pandemic halted clinical research. As a measure of outcome quality of this alignment, we averaged DFNS-standardized z-scores across all control subjects: the resulting mean was −0.03 (DFNS 95% CI 0.01 ± 0.25) and SD was 1.26 (DFNS 95% CI 0.99 ± 0.10). Thus, Boston (where CON were collected) had no systematic bias towards overestimating or underestimating QST parameters, but a larger variance than the original 10 sites of DFNS.^40^

Although researchers tried to stay naïve to the participant group, many participants disclosed information about their pain during the study visit.

Quantitative sensory testing was applied to the lower abdomen or pelvis, below the umbilicus. Specifically, thermal, pinprick, and von Frey stimuli were delivered to the skin of the lower abdomen in varied locations to assess general sensation in the area and to avoid surgical scars, pressure pain threshold was measured on the muscles of the lower abdominal wall, and vibration detection was measured on the symphysis pubis (see Table 1 for more information on QST measures).

**Table 1:** Quantitative sensory testing abbreviations and methods.

Abbreviation	Full name	Method
CDT	Cold detection threshold	Cooled from baseline until participant senses “cooling.” Three repetitions are performed at each site. Arithmetic mean of change in temperature is used in analysis
WDT	Warm detection threshold	Warmed from baseline until participant senses “warming.” Three repetitions are performed at each site. Arithmetic mean of change in temperature is used in analysis
TSL	Thermal sensory limen	Warmed and cooled from baseline asking participant to indicate when they feel a change. Calculated by subtracting the arithmetic mean of the cool detections from the arithmetic mean of the warm detections during the alternations
PHS	Paradoxical heat sensation	During TSL, if participants report “warm” or “hot” sensations during cooling, this is a paradoxical heat sensation. These are counted, and there is a maximum of 3
CPT	Cold pain threshold	Cooled from baseline until participant feels pain sensation. Three repetitions are performed at each site. Arithmetic mean of absolute temperatures is used in analysis
HPT	Heat pain threshold	Heated from baseline until participant feels pain sensation. Three repetitions are performed at each site. Arithmetic mean of absolute temperatures is used in analysis
MDT	Mechanical detection threshold or tactile detection threshold	Using von Frey hairs the tactile detection threshold is determined by performing a modified method of limits. Five threshold determinations are made, each with a series of ascending and descending stimulus intensities. The final threshold is the geometric mean of these 5 series of suprathreshold and subthreshold stimuli intensities
MPT	Mechanical pain threshold	This test uses weighted pinprick stimuli. Five threshold determinations are made, each with a series of ascending and descending stimulus intensities. The final threshold is the geometric mean of the 5 suprathreshold and subthreshold readings (modified method of limits)
MPS	Mechanical pain sensitivity	To test for mechanical pain sensitivity, weighted pinprick stimuli of different stimulus intensities are used so that a stimulus–response function is obtained for pinprick-evoked pain (Numerical Rating Scale; range 0-100). Seven stimuli intensities are applied 5 times each at both test sites in a randomized order, during which the subject is asked to give a numerical pain rating immediately after each stimulus. The degree of pain sensitivity is calculated by the geometrical mean of the pain ratings given for pinprick stimuli (MPS)
DMA	Dynamic mechanical allodynia	Dynamic mechanical allodynia is tested by using the same test pattern as described for the MPS. Dynamic innocuous stimuli (Q-tip, cotton wisp, and soft brush) are applied in between the pinprick stimuli in a randomized order. Each of the 3 innocuous stimuli is tested 5 times on each test site. The degree of pain sensitivity is calculated by the geometrical mean of the pain ratings innocuous stimuli (DMA)
WUR	Wind-up ratio	The numerical pain rating (NRS; range 0-100) given for an applied series of repetitive pinprick stimuli of the same intensity (10 stimuli with a repetition rate of 1/s, 256 mN) is compared with the numerical pain rating of a single stimulus again of the same intensity. This procedure is repeated 5 times. A “wind-up” ratio is calculated by the arithmetic mean of the pain intensity rating for the series of stimuli divided by the arithmetic mean of the pain intensity rating for the single stimulus
VDT	Vibration detection threshold	This test is performed with a standardized tuning fork (64 Hz) that is placed over a bony prominence. The vibration detection threshold is determined by 3 series of descending stimulus intensities determined from the “wandering” tip of a triangle moved by means of the vibration and indicated on the tuning fork^29^ using the arithmetic mean of the values when the participant just stopped perceiving vibration (in x/8)
PPT	Pressure pain threshold	Using a pressure algometer (contact area 1 cm^2^), the threshold for pressure induced pain is measured above a muscle in 3 series of slowly increasing stimulus intensities (0.5 kg/s, corresponding to ca. 50 kPa/s). The threshold is then determined as the arithmetic mean of the 3 series (in kPa).

Here are the abbreviations used for each QST measure in the DFNS protocol and the method by which they are collected. Methods are adapted from DFNS QST Investigator's Brochure Version 2.1.^50,56^

### 2.3. Data analysis

All data were collected using the official QST form and manually uploaded to a secure database. Data inputting was independently verified. Data were analysed as per protocol,^40,54^ using MATLAB (R2021a) for data analysis and Prism9 to create figures. Published reference data were used to Z transform the data for the foot.^54^ Published reference data are not available for the abdomen or pelvis, and therefore, after discussion with the DFNS (R.-D.T. and J.V.), published reference data for the back were used.^51^ A Z-score greater than 0 shows a gain of function, and a Z-score less than 0 shows a loss of function.

In addition, statistical comparisons were made between the pain groups (EAP, EABP, BPS, and PP) and our control group (CON). These comparisons were performed with Student *t* tests using the Z-transformed data. Multiple comparison correction was performed using Bonferroni correction, when looking at individual QST measures corrections were applied to QST “blocks” of thermal detection, thermal pain thresholds, mechanical pain thresholds, and mechanical detection.

When comparing painDETECT^23,24^ scores for individual questions, correlations with the relevant QST block were performed (ie, painDETECT question “is cold or heat (bath water) in this area occasionally painful?” correlated with thermal pain QST measures). Normality tests were performed on each painDETECT sensation variable (ie, those scored out of 5, excluding question on time course and spatial properties of the pain) and appropriate tests were used. Paraesthesia symptoms “burning” and “tingling” from painDETECT do not have comparators within QST so have been excluded from this analysis. In addition, the proportion of those reporting clinically significant painDETECT scores for sensations are reported (ie, are any responses >3 of 5, representing strongly or very strongly). To account for interindividual differences in pain sensitivity, painDETECT scores for each of these symptoms were recalculated by subtracting the mean across all 7 responses from each individual response^9^ when comparing scores between groups. Scores larger than zero thereby indicate a sensation that is more intense than the average individual symptom score.

Other variable scores such as pain intensity (Numerical Rating Scale [NRS] 0 to 10) and state anxiety (State-Trait Anxiety Inventory STAI-S^58^) were normality tested and are reported appropriately.

For menstrual cycle stage, participants were asked to self-report whether they were taking any hormonal contraceptives, the day of their last menstrual period, and typical length of their menstrual cycle. Those who were not currently taking any form of hormonal contraception, who indicated that they still had menstrual cycles, were categorised by menstrual phase according to the following protocol: based on a 28-day cycle, day 1 to 7 were classified as a menstrual phase, day 8 to 14 were classified as follicular or proliferative and day 15+ was the luteal or secretory phase. For participants whose cycle length deviated from 28 days, 14 days were subtracted from their reported cycle length, the secretory phase being held constant, and the remaining duration was assigned to the proliferative phase. For women who reported a variable cycle length, the min, mean, or max cycle length was determined, and stage was allocated accordingly. The menstrual phase was cross-checked by 2 researchers (L.C. and D.P.) to ensure consistency.

To determine whether individuals with CPP (EAP, BPS, EABP, and PP combined) could be categorised into the clusters previously described,^8^ a recently developed algorithm^64^ was used. The clusters are as follows: ‘healthy,’ ‘sensory loss,’ ‘thermal hyperalgesia’ and ‘mechanical hyperalgesia.’ We used a deterministic approach such that participants were sorted into the cluster which they had the greatest score for, based on their abdominal sensory profiles. To assess differences between these clusters in questionnaire measures, analysis of variance or Kruskall–Wallis test was used according to normalcy.

## 3. Results

### 3.1. Demographics

Eighty-five women were recruited and underwent the full QST profiling (EAP n = 25, BPS n = 13, EABP n = 15, PP n = 6, and CON n = 26). Overall, most of the pain groups were well matched for age, menstrual cycle phase, and state anxiety scores (Table 2); however, the BPS group were significantly older than the CON and PP groups (*P* = 0.010 and *P* = 0.090, respectively) and significantly more anxious than the CON group (*P* = 0.009). The EAP, BPS, and EABP had significantly higher current pain intensity NRS scores than CON (all *P* < 0.01), see Table 2. 66.7% of participants were taking hormones, with the remainder being spread across menstrual cycle phases (Table 2) (for n = 1 no data on hormones or cycle were available and for n = 5 participants they were not on hormones, but it was not possible to determine the menstrual stage).

**Table 2:** Participant characteristics.

	EAP	BPS	EABP	PP	CON
No. of participants	25	13	15	6	26
Age	34 (22-50)	46 (27-51)	31 (20-51)	31 (25-34)	29.5 (21-45)
Current pain intensity	1 (0-6)	3 (0-8)	2 (0-7)	0 (0-2)	0 (0-2)
Duration of pain (y)	18 (7-33)	19 (0-37)	21 (6-35)	17 (11-22)	—
painDETECT score	9.2 (0-18)	14.6 (2-29)	14 (7-26)	9 (3-13)	N/A
painDETECT neuropathic, n (% of group)	0 (0)	5 (41.7)	3 (23.1)	0 (0)	N/A
painDETECT mixed n (% of group)	4 (23.5)	1 (8.3)	5 (38.5)	1 (16.7)	N/A
painDETECT nociceptive n (% of group)	13 (76.5)	6 (50)	5 (38.5)	5 (83.3)	N/A
State anxiety score	32.1 (20-49)	41 (20-67)	33 (22-56)	28.5 (20-43)	26 (20-41)
Menstrual phase n (% of group)					
Menstrual	2 (8)	0 (0)	0 (0)	1 (16.7)	4 (16)
Proliferative	1 (4)	0 (0)	1 (6.7)	0 (0)	4 (16)
Secretory	2 (8)	1 (8.3)	2 (13.3)	2 (33.3)	3 (12)
Taking steroid hormones n (% of group)	20 (80)	9 (75)	12 (80)	3 (50)	12 (48)
Medications n (% of group)					
NSAIDS	1 (4)	0 (0)	2 (13.3)	1 (16.7)	1 (3.8)
Other over-the-counter painkillers (eg, paracetamol)	3 (12)	1 (7.7)	3 (20)	0 (0)	0 (0)
Antidepressants/Anxiolytics	8 (32)	6 (46.2)	7 (46.7)	2 (33.3)	6 (23.1)
Medications for other health conditions (eg, allergy medication)	15 (60)	6 (46.2)	9 (60)	2 (33.3)	12 (46.2)
Vitamins/minerals/probiotics/herbal	5 (20)	1 (7.7)	2 (13.3)	0 (0)	7 (26.9)
Opioids	1 (4)	0 (0)	1 (6.7)	0 (0)	0 (0)
Muscle relaxants	1 (4)	2 (15.4)	0 (0)	0 (0)	0 (0)
Gabapentinoids	1 (4)	0 (0)	1 (6.7)	0 (0)	0 (0)
Surgery to diagnose/exclude endometriosis n (% of group)	25 (100)	0 (0)	14 (100)	2 (33.3)	0 (0)
Stage of endometriosis n (% within those who have had surgery to diagnose/exclude endometriosis)					
Stage I	9 (36)	0 (0)	7 (46.7)	0 (0)	—
Stage II	4 (16)	0 (0)	3 (20)	0 (0)	—
Stage III	2 (8)	0 (0)	1 (6.7)	0 (0)	—
Stage IV	6 (24)	0 (0)	2 (13.3)	0 (0)	—
Stage unknown	4 (16)	0 (0)	2 (13.3)	0 (0)	—
No endometriosis	0 (0)	0 (0)	0 (0)	0 (0)	—

Shown are participant demographics for each of the TRiPP subgroups: EAP (endometriosis-associated pain), BPS (bladder pain and urinary symptoms), EABP (comorbid endometriosis and bladder symptoms), PP (pelvic pain without endometriosis or bladder symptoms), and CON (controls without pelvic pain or endometriosis). Age is given in years with mean and range. Pain intensity is given as median score on 0 to 10 NRS scale with range. Duration of pain is given in years as a median score with range; for the CON group, this is not applicable as they are pain free. Current state anxiety score is given as a mean and range based on State-Trait Anxiety Inventory—State Questionnaire,^60^ scores of 20 to 39, 40 to 59, and 60 to 80 indicate low, moderate, and high anxiety, respectively. painDETECT^25^ score is given as mean and range. Medications taken in the 24 hours before the study visit, as broken down to medication groups are shown as counts. Surgery to diagnose or exclude endometriosis is given as counts and percentages per group. The stage of endometriosis shown as count and percentage of those who had received surgery to diagnose or exclude endometriosis.

Table 3 shows the proportion of participants that reported clinically significant sensory symptoms on the painDETECT measure. For the endometriosis groups (EAP and EABP), pain attacks was the most common symptom, whereas for the BPS groups ongoing burning pain was the most common and this was also common in the comorbid group EABP. Patients with bladder pain (BPS and EABP) had more frequent pressure-evoked pain than EAP or PP.

**Table 3:** Reported symptoms from painDETECT.

Sensory symptom	EAP (n = 24)	EABP (n = 15)	BPS (n = 13)	PP (n = 6)
Burning	8.3%	33.3%	69.2%	0%
Prickling	0%	6.7%	30.8%	16.7%
Mechanical allodynia	4.2%	0%	7.7%	0%
Painful attacks	37.5%	53.3%	23.1%	16.7%
Thermal hyperalgesia	4.2%	0%	0%	0%
Numbness	4.2%	6.7%	0%	0%
Pressure-evoked pain	12.5%	33.3%	23.1%	0%

Proportion of participants in each pain group reporting clinically significant symptoms (ie, a score >3, strongly or very strongly) in the painDETECT questionnaire.^25^

BPS, bladder pain syndrome; EABP, endometriosis-associated pain with comorbid bladder pain; EAP, endometriosis-associated pain; PP, pelvic pain.

One participant in the EAP group reported experiencing pain on the right foot on the day of testing; therefore, their control site data are excluded from all analysis.

### 3.2. Quantitative sensory testing profiles

**Figure 1.**
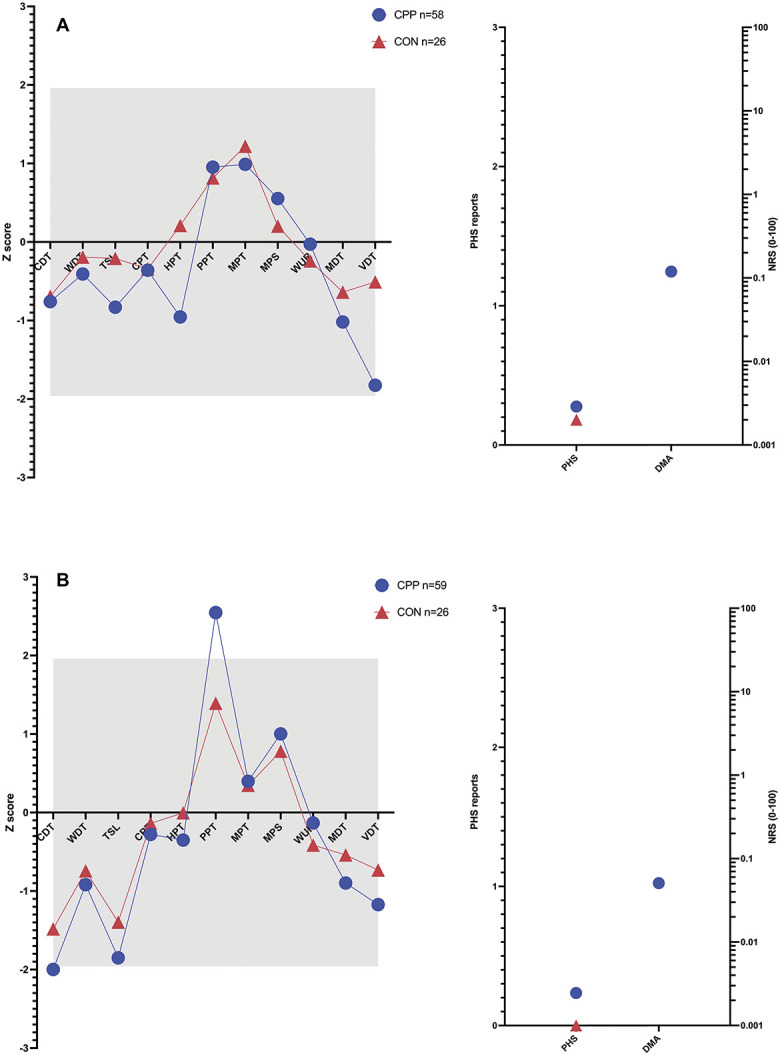
(A) Sensory profiles at the foot control site and (B) sensory profiles at the abdomen. CDT, cold detection threshold; CON group, controls without pain; CPP, participants with chronic pelvic pain; CPT, cold pain threshold; DMA, dynamic mechanical allodynia (DMA ratings do not occur in healthy people (published reference data) or in our CON group); HPT, hot pain threshold; MDT, mechanical detection threshold; MPS, mechanical pain sensitivity; MPT, mechanical pain threshold; PHS, paradoxical heat sensation; PPT, pressure pain threshold; TSL, thermal sensory limen; VDT, vibration detection threshold; WDT, warm detection threshold; WUR, wind up ratio.

Figure 1 shows the sensory profiles at the foot control site (Fig. 1A) and the lower abdomen test site (Fig. 1B) for the CON compared with those with CPP. There are significant differences between the CPP and CON groups for: thermal sensory limen at both the foot and abdomen (t= −2.8, *P* = 0.032 and t= −3.5, *P* = 0.004, respectively), suggesting loss of small fibre function in CPP, vibration detection at the foot (t = −3.0, *P* = 0.017) indicating large fibre defect in CPP, and pressure pain threshold at the abdomen (t = 3.0, *P* = 0.012) showing large gain of function in CPP suggestive of central sensitization to deep tissue input.

**Figure 2.**
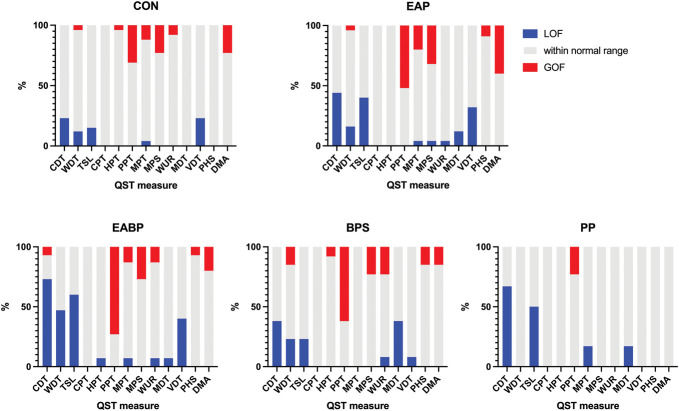
Heterogeneity within and between TRiPP subgroups as shown by the distribution of loss of function and gain of function to QST measures at the abdomen test site. Shown in red are the proportion of the group which have gain of function relative to the normal range from reference data. Shown in grey are those within the normal range. Shown in blue is the proportion showing loss of function. BPS, bladder pain; CDT, cold detection threshold; CON, pain-free controls; CPT, cold pain threshold; DMA, dynamic mechanical allodynia; EABP, comorbid endometriosis and bladder pain; EAP, endometriosis-associated pain; HPT, heat pain threshold; MDT, mechanical detection threshold; MPS, mechanical pain sensitivity; MPT, mechanical pain threshold; PHS, paradoxical heat sensation; PP, pelvic pain without endometriosis or bladder symptoms; PPT, pressure pain threshold; QST, quantitative sensory testing; TSL, thermal sensory limen; VDT, vibration detection threshold; WDT, warm detection threshold; WUR, wind up ratio.

Figure 2 shows the heterogeneity within subgroups showing the proportion with “normal” function, gain of function, and loss of function for each test variable, as well as illustrating differences within the CPP cohort divided into the TRiPP subgroups. At the test site (low abdomen), there was loss of function in thermal detection (indicating small fibre loss), as well as gain of function for pressure pain thresholds, mechanical pain thresholds, mechanical pain sensitivity, or increased dynamic mechanical allodynia across groups.

At the test site, compared with our CON group, those with bladder pain (EABP and BPS combined) showed significant gain of function for pressure pain threshold (t = 3.7, *P* = 0.003), suggesting central sensitization to deep tissue input as well as loss of function for thermal sensory limen (TSL) (t = −3.0, *P* = 0.02), suggesting loss of small fibre function. However, those with endometriosis (EAP and EABP combined) had significant loss of function for TSL at both the test (t = −3.5, *P* = 0.003) and control site (t = −3.2, *P* = 0.008) (ie, small fibre loss). In the PP compared with CON groups, there was greater dynamic mechanical allodynia (t = −10.7, *P* < 0.001). However, it should be remembered that this was a small group (n = 6). All other measures were within the 95% confidence intervals from the reference data (shown by grey box).

### 3.3. Quantitative sensory testing clusters

**Figure 3.**
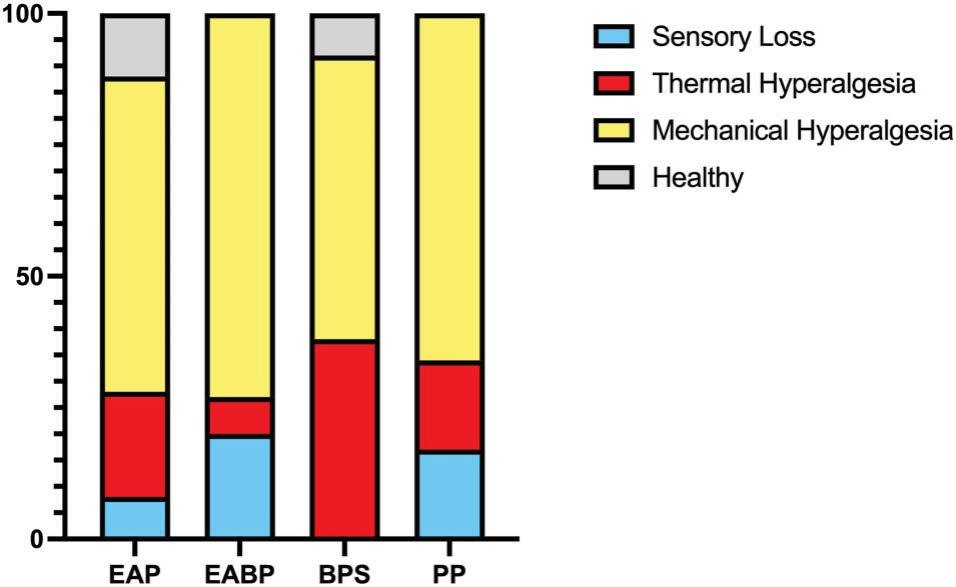
Cluster allocation by group. Sensory phenotypes of sensory loss, thermal hyperalgesia, mechanical hyperalgesia, and healthy determined by published algorithm^70^ based on individual QST profiles. BPS, bladder pain group; EABP, comorbid endometriosis and bladder pain; EAP, endometriosis-associated pain group; PP, pelvic pain without endometriosis or bladder symptoms; QST, quantitative sensory testing.

All 4 previously identified QST clusters were present in the cohort of women with CPP. Profiles were consistent with those of healthy subjects in only a small number (6.8%) of women with CPP; thus, more than 93% had QST sensory profiles suggesting some altered somatosensory processing. Cluster allocation for the TRiPP subgroups (EAP, BPS, EABP, and PP) are shown in Figure 3. For all TRiPP subgroups, the most common cluster allocation was “mechanical hyperalgesia” with at least 50% of participants in each group fitting this cluster, which suggests some central sensitization.

There was no significant difference between the clusters for age (*P* = 0.52), pain intensity rating (NRS 0-10 at the time of testing) (*P* = 0.72), state anxiety (*P* = 0.40), or painDETECT score (*P* = 0.23).

### 3.4. Comparison with painDETECT scores

Correlations between sensory descriptors from painDETECT and relevant QST measures are shown in Table 4. Significant correlations were found between the descriptor “does slight pressure in this area, eg, with a finger, trigger pain?” (pressure-evoked pain) and the pressure pain threshold (*r* = 0.47, *P* < 0.001) and “is cold or heat (bath water) in this area occasionally painful?” (thermal hyperalgesia) and the heat pain threshold (*r* = 0.32, *P* = 0.032). The item “is light touching (clothing, a blanket) in this area painful?” (mechanical allodynia) was strongly correlated with mechanical pain sensitivity (*r* = 0.38, *P* = 0.009) but not with dynamic mechanical allodynia (*r* = 0.12, *P* = 0.378 uncorrected); this item may reflect skin hypersensitivity but not necessarily to light touch.

**Table 4:** Correlations between relevant painDETECT measures and quantitative sensory testing measures.

QST measure	painDETECT question	*r*	*P*
CPT	Is cold or heat (bath water) in this area occasionally painful?	0.25	0.12
HPT	Is cold or heat (bath water) in this area occasionally painful?	0.32*	0.032*
VDT	Do you suffer from a sensation of numbness in the area?	−0.28	0.036 (uncorrected)
MDT	Do you suffer from a sensation of numbness in the area?	−0.20	0.28
PPT	Does slight pressure in this area, eg, with a finger, trigger pain?	0.47*	<0.001*
MPT	Is light touching (clothing, a blanket) in this area painful?	0.03	0.85 (uncorrected)
MPS	Is light touching (clothing, a blanket) in this area painful?	0.38*	0.009*
DMA	Is light touching (clothing, a blanket) in this area painful?	0.12	0.38 (uncorrected)

Shown are *r* values from Pearson correlation and *P* values which are corrected for multiple comparisons (using Bonferroni correction) unless otherwise stated. painDETECT questions relate to clinical symptoms: “Is cold or heat (bath water) in this area occasionally painful?” relates to thermal hyperalgesia; “do you suffer from a sensation of numbness in the area?” relates to numbness; “does slight pressure in this area, eg, with a finger, trigger pain?” relates to pressure evoked pain; “is light touching (clothing, a blanket) in this area painful?” relates to mechanical allodynia.

* Significance *P* < 0.05.

CPT, cold pain threshold; DMA, dynamic mechanical allodynia; HPT, hot pain threshold; MDT, mechanical detection threshold; MPS, mechanical pain sensitivity; MPT, mechanical pain threshold; PPT, pressure pain threshold; VDT, vibration detection threshold.

In addition, there was a significant difference between the QST clusters described above and individually mean adjusted scores for painDETECT variables: numbness (F = 3.71, *P* 0.017) and mechanical allodynia (F = 3.15, *P* = 0.032). Post hoc tests showed for numbness a significant difference between sensory loss and thermal hyperalgesia clusters (t = 2.93, *P* = 0.018, uncorrected) as well as between thermal hyperalgesia and healthy (t = −2.255, *P* = 0.041, uncorrected), although these did not withstand multiple comparison correction. For mechanical allodynia, there were significant differences between sensory loss and thermal hyperalgesia clusters (t = −4.10, *P* = 0.006), sensory loss and mechanical hyperalgesia (t = −5.88, *P* = 0.0006), and sensory loss and healthy (t = −2.50, *P* = 0.032, uncorrected).

## 4. Discussion

This is the first study using the comprehensive DFNS QST protocol for profiling and phenotyping women with CPP.^40,54^ When looking at the abdominal site, we see gain of function in pressure pain thresholds suggesting changes in pain processing pathways to deep tissue input. We also see loss of function in thermal detection thresholds, suggesting loss of small fibre function. However, when divided into clinically defined subgroups (ie, by the presence of endometriosis or bladder pain), more nuanced differences are seen, specifically a gain of function for pressure pain thresholds and a loss of function for heat pain thresholds for those with bladder pain and a gain of function for dynamic mechanical allodynia for those with endometriosis. Both those with endometriosis or bladder pain showed loss of function for thermal sensory limen (detecting changes from warm to cool). There is, however, marked heterogeneity in the sensory profiles seen even within clinically defined subgroups, and this is illustrated by the observation that all 4 sensory phenotypes are represented. Mechanical hyperalgesia seems to be the most common sensory phenotype across all clinical subgroups. Notably, only 6.8% of CPP have “healthy” sensory function.

### 4.1. Mechanisms of pain

Traditionally, pelvic pain has been considered predominantly a visceral pain condition^5^ although increasing evidence suggests that there is frequently a central component.^33^ Given alterations in sensory function found at the abdomen or pelvis, our data suggest that some women with CPP (regardless of underlying pathology) have changes to peripheral nerve function. These changes could be the result of neuropathic-like pain, which the painDETECT scores suggest could be present in this cohort.^12,23,24^ Alternatively, these could be due to nociplastic or central mechanisms.^4,21,36,37,71^

The most striking observation is gain of function in PPT. Although there was only a statistically significant difference between CON and those in EABP and BPS groups, Figure 2 illustrates that across all the CPP groups there is a large proportion demonstrating a gain of function in PPT. Although it is not possible to determine from our data how this change is generated, the association with those with bladder pain and urinary symptoms particularly suggests that there may be a potential role for referred hyperalgesia secondary to viscero–somatic communication.^74,76^

The data also showed significant gain of function in DMA in 22.5% of the participants with endometriosis (EAP and EABP) (as seen in Fig. 2) which is consistent with studies describing allodynia on the abdomen.^48,60^ We can only speculate about factors generating this observation; however, it is important to remember the role of laparoscopy in both the diagnosis and treatment of endometriosis and the investigation of CPP more broadly.^17,34,49^ All participants in EAP and EABP groups, and some of the BPS group, were initially recruited into parent studies at the time of surgery, and many will have had multiple surgeries both for their pelvic pain and for other indications (eg, acute appendicitis or caesarean sections). These surgeries carry the risk of postoperative pain, localised hyperexcitability, and numbness.^22,52,57,62^

The gain of function in pressure pain thresholds suggest peripheral or central sensitization to deep input, whereas mechanical pain measures (MPT, MPS, and DMA) suggest central sensitization to cutaneous input.

Importantly, we also see loss of function in response to stimuli across all subgroups of CPP. Although hyperalgesia and allodynia have been reported in CPP,^3,28,30,31,56^ such loss of function markers may suggest different mechanisms which could play an important role in understanding CPP. This loss of function could be due to “deafferentation” as has been seen in other conditions.^8,29,65^ Alternatively, it could be due to “descending defunctionalization” of nonnociceptive somatosensory processing, as has been suggested in neuropathic pain conditions where sensory loss is seen on ipsilateral and contralateral areas.^18^

The sensory manifestations are not consistent across women, either when considering the cohort as a whole or dividing into clinically determined subgroups. This is, however, consistent with the known heterogeneity of CPP^2,11,77^ and may contribute to the variation seen in the efficacy of standard treatments.^25,77^ Strategies to subgroup women based on their somatosensory profiles (and possible underlying mechanisms) could be of enormous benefit in this context.

We explored whether mechanistically relevant clusters could be identified in the present cohort as in other patient groups.^8,64,70^ The strategy applied^70^ identifies 4 subgroups in patients with neuropathic pain, believed to represent (1) those with irritable nociceptors^14,20^ (thermal hyperalgesia), (2) deafferentation^10,20,65^ (sensory loss), (3) central sensitization^10,20^ (mechanical hyperalgesia), and (4) those with normal peripheral nerve function. Although it remains to be seen if these clusters respond differently to treatment,^8,69,70^ there is preliminary evidence suggesting they will^7^ and differences in pain interference between these clusters have been shown.^27^ We were able to identify women fitting each of these clusters, suggesting that the 3 mechanistically different sensory abnormal groups are relevant to CPP.

The commonest sensory phenotype allocation was “mechanical hyperalgesia.” Human surrogate models of known central sensitization in the spinal cord had this pattern, in particular mechanical pain measures (MPT, MPS, and DMA); some here may, therefore, have spinal long-term potentiation.^55^ On the other hand, gain of function for pressure pain threshold has been seen in fibromyalgia,^43,46^ complex regional pain syndrome,^42,66^ and after sleep deprivation^59^ or other stressors^44,67^; although still central sensitization, the neuronal populations involved may be different. The “thermal hyperalgesia” phenotype seen in the BPS group particularly may represent the concept of irritable nociceptors.^8^

### 4.2. Clinical relevance

Chronic pelvic pain is challenging to treat; therefore, it is important to better understand the underlying pain mechanisms and to identify strategies to determine who might respond to specific treatments. The present findings highlight that dysfunction in somatosensory processing pathways is present for many women with CPP no matter the underlying cause. Importantly, we found that >93% of our cohort are classified as having altered somatosensory nervous system function, yet clinically medications targeting peripheral or central somatosensory signalling are not routinely used. This disconnect needs to be addressed to improve patient care.

The sensory phenotypes from QST used in this study represent one potential stratifier; however, the utility of QST clinically is currently limited due to time, equipment and/or training requirements. Although there are ongoing efforts to create simple, clinically accessible, bedside QST tools,^38,53,72^ the use of patient-completed questionnaires would be even cheaper and more efficient. Although studies have had mixed results when trying to find tight parallels between QST and painDETECT previously,^26,32,47,63,73^ this study shows correlations between factors assessing particular sensory phenomena. Notably PPT assessed using QST, which clearly showed a gain of function in many participants with CPP, showed strong correlation with the response to the relevant painDETECT question (“does slight pressure in this area, eg, with a finger, trigger pain?”). Interestingly, however, numbness was not correlated with loss of function measures, and this may be an important limitation of this approach. Further work is needed to determine both the utility and acceptability of using painDETECT as a component of clinical and research assessments of CPP in situations where QST is not feasible.

### 4.3. Limitations

Attempts were made to ensure consistency of data by using the same equipment at all 3 sites and training all personnel together in data collection methods. Although the overall sample size is comparable with other QST studies,^35,39,50^ when divided into clinical subgroups particularly the PP group is too small to meaningfully interpret results. Where appropriate, comparisons were made between those with and without endometriosis and those with and without bladder pain.

In addition, we cannot exclude that some of the differences we found were based in site differences of this multisite study. This is based in the respective centre's expertise and clinical focus. A future study should aim to include recruiting at centres covering all groups equally.

The lower abdomen or pelvis test site was selected as the most clinically meaningful area as this is often the referred pain site in CPP patients. However, there is no published reference QST data from this site; we, therefore, used published QST data from the trunk area.^51^ The study compared patient data with a control group (CON), and data were z-transformed using the trunk reference data. We did not consider it appropriate to use the control group as reference data for z-transformation as the sample size is not large enough and the spread of ages is relatively limited.

## 5. Conclusions

The present multicentre study showed significant changes of somatosensory function in women with CPP, with 6.8% showing a “healthy” sensory profile. Specific sensory alterations are present across different underlying pathologies, whereas others are disease specific. Our findings suggest that there is central sensitization to deep and cutaneous inputs in women with CPP, in addition to a variety of alterations in peripheral nerve function. The data showed a moderate correlation between QST measures and relevant descriptors from painDETECT, suggesting painDETECT may have utility for phenotyping CPP patients in specific settings. Understanding somatosensory processing may be helpful for phenotyping CPP patients. Stratification methods such as these may in future guide personalised pain management and thus should be considered when designing clinical trials.

## Conflict of interest statement

L. Coxon: no competing interests. J. Vollert received consulting fees from Vertex Pharmacauticals, Embody Orthopaedic, and Casquar. D. Perro receives financial support from the Canadian Institute of Health Research Doctoral Foreign Study Award. C. E. Lunde: no competing interests. J. Ferreira-Gomes: no competing interests. A. Charrua: no competing interests. P. Abreu-Mendes: no competing interests. J. Birch: no competing interests. J. Meijlink: member of scientific advisory board for Glycologix. L. Hummelshoj: no competing interests. A. Hoffmann: employee of Bayer AG, Germany. Q. Aziz: no competing interests. L. Arendt-Nielsen: no competing interests. E. Pogatzki-Zahn received financial support from Grunenthal and Mundipharma for research activities and advisory and lecture fees from Grünenthal, Novartis, and Mundipharma. In addition, she receives scientific support from the German Research Foundation (DFG), the Federal Ministry of Education and Research (BMBF), the German Federal Joint Committee (G-BA), and the Innovative Medicines Initiative (IMI) 2 Joint Undertaking under grant agreement No 777500. This joint undertaking receives support from the European Union's Horizon 2020 research and innovation programme and EFPIA. All money went to the institution E. Pogatzki-Zahn is working for. M. Krassowski: no competing interests. E. Evans: no competing interests. L. Demetriou: no competing interests. S. A. Missmer has been an advisory board member for AbbVie and Roche and receives research funding from the National Institutes of Health, the US Department of Defense, the J. Willard and Alice S. Marriott Foundation, and AbbVie; none are related to the presented work. The J. Willard and Alice S. Marriott Foundation supported enrollment of and data collection from the A2A cohort in Boston from which TRiPP data were sampled. C. M. Becker: Research Grants from Bayer Healthcare, MDNA Life Sciences, Roche Diagnostics, European Commission, and NIH. His employer has received consultancy fees from Myovant and ObsEva for work outside of this project. K. T. Zondervan: reports grant funding from EU Horizon 2020, NIH US, Wellbeing of Women, Bayer AG, Roche Diagnostics, Evotec-Lab282, and MDNA Life Sciences, outside the submitted work. A. W. Horne reports grant funding from the MRC, NIHR, CSO, Wellbeing of Women, Roche Diagnostics, Astra Zeneca, Ferring, Charles Wolfson Charitable Trust, and Standard Life. His employer has received consultancy fees from Roche Diagnostics, AbbVie, Nordic Pharma, and Ferring, outside the submitted work. In addition, A. W. Horne has a patent for a serum biomarker for endometriosis pending. C. B. Sieberg: no competing interests. F. Cruz: consultant, speaker, or investigator for Allergan (Abbvie), Astellas, Bayer, Ipsen, and Recordati. R.-D. Treede: Ad board for BAYER, IASP task force on chronic pain classification. J. Nagel: employee and shareholder of Bayer AG, Germany. K. Vincent declares research funding from Bayer Healthcare and honoraria for consultancy and talks and associated travel expenses from Bayer Healthcare, Grunenthal GmBH, AbbVie, and Eli Lilly.
